# Evaluation of a Hybrid Approach Using UBLAST and BLASTX for Metagenomic Sequences Annotation of Specific Functional Genes

**DOI:** 10.1371/journal.pone.0110947

**Published:** 2014-10-27

**Authors:** Ying Yang, Xiao-Tao Jiang, Tong Zhang

**Affiliations:** Environmental Biotechnology Laboratory, Department of Civil Engineering, The University of Hong Kong, Hong Kong SAR, China; Hellas, Greece

## Abstract

The fast development of next generation sequencing (NGS) has dramatically increased the application of metagenomics in various aspects. Functional annotation is a major step in the metagenomics studies. Fast annotation of functional genes has been a challenge because of the deluge of NGS data and expanding databases. A hybrid annotation pipeline proposed previously for taxonomic assignments was evaluated in this study for metagenomic sequences annotation of specific functional genes, such as antibiotic resistance genes, arsenic resistance genes and key genes in nitrogen metabolism. The hybrid approach using UBLAST and BLASTX is 44–177 times faster than direct BLASTX in the annotation using the small protein database for the specific functional genes, with the cost of missing a small portion (<1.8%) of target sequences compared with direct BLASTX hits. Different from direct BLASTX, the time required for specific functional genes annotation using the hybrid annotation pipeline depends on the abundance for the target genes. Thus this hybrid annotation pipeline is more suitable in specific functional genes annotation than in comprehensive functional genes annotation.

## Introduction

In recent years, the rapid development of next generation sequencing (NGS) has broadened the application of metagenomics in various aspects of biological research [1]. The reduction of DNA sequencing cost has surpassed the rate predicted by Moore's law [2]. The nosedive of sequencing cost per nucleotide resulted in the exponential growth of NGS data. More NGS sequences were generated in the 1000 genomes project within its first 6 months than the sequence data accumulated in NCBI Genbank database over two decades [3].

The deluge of NGS data poses higher requirement on computational resource for data analysis, which became the bottleneck for metagenomic analysis other than the sequencing cost. For example, illumina NextSeq 500 is able to generate 30–120 Gb of microbiological metagenomic data within 30 hours. But it may take months to analyze these data, for annotation of the overall functions of these genes. Besides the time cost of metagenomic analysis, cost of computational resources is getting higher for handling the overwhelming increase of data generated, not to mention the hardly quantifiable human resources needed for metagenomic data analysis currently [2].

The common analysis of metagenomic data included annotation, assembly [4], and genome binning [5]. Annotation of metagenomic sequences is one of the most fundamental analyses to extract taxonomy composition and functional information. Functional annotation is mostly performed by similarity search or mapping of data sequences against a reference protein database using various algorithms (see the review of Scholz et al. [6]). BLASTX [7] is widely applied in sequences alignments because it is more sensitive and could be used to find distant homologous sequences in annotation using database search [6]. However, running a BLASTX similarity search for functional annotation is computational intensive and time consuming in terms of CPU time, as much as ten times higher than the cost of sequencing [1]. Efforts have been made to develop ultra-fast tools which can be used for aligning metagenomic sequences against a reference database based on homology search, such as BLAT [8], RAPSearch [9], the upgraded RAPSearch2 [10] and UBLAST [11]. The drawback of these ultra-fast tools is that some hits might be missed. The portion of missed BLASTX hits is about 1.3–3.2% using RAPSearch [9] and the portion of missed hits using BLAT can reach to 20% [10]. The pre-developed Hidden Markov Models (HMMs) is another fast approach for finding conserved domains. However, the limitation of HMMs is that it does not function very well for short reads [6].

Besides the development of ultra-fast tools for database search, efforts have been made to shorten the analysis time through constructing new specific databases and optimizing the existing database as well. For function analysis in metagenomics study, the NCBI-nr database is one of the most common databases. It contains both metabolic pathway information, and functionally related taxonomic information. However, it would be a waste of time and computer resources to align the whole set of metagenomic sequences to NCBI-nr database (or other general databases, like KEGG) for specific function or metabolic pathway study, such as antibiotic resistance genes (ARGs) and nitrification related genes, since genes of these specific functions only account for a very small portion in the whole NCBI database. Using specific databases instead of the whole NCBI database is a more efficient approach although it needs further validation through a two-step pipeline [12]. Among a few ARGs databases constructed [13], [14], the Antibiotic Resistance Genes Database (ARDB) has been optimized by removing error sequences and duplicate sequences to shorten the BLASTX time [15]. But the BLASTX time against this optimized ARDB database is still long, especially for large datasets.

A previous study proposed a hybrid approach for taxonomic annotation [16]. It used multiple alignment processes (UBLAST and BLASTX) to accelerate homology search and achieve rapid identification of taxonomic assignments for metagenomic data. In the present study, we first compared the time consumed and annotation results of two ultra-fast alignment tools, i.e. RAPSearch2 and UBLAST in ARGs annotation. Then we evaluated the hybrid approach which uses UBLAST and BLASTX to achieve fast functional annotation of metagenomic sequences for specific functional genes. UBLAST is used for ultra-fast identification of potential matched sequences and BLASTX is applied for more accurate identification and final annotation of the target sequence from the potential matched sequences selected by UBLAST. This hybrid pipeline was demonstrated very efficient and accurate for sequence annotation using specific functional protein database, such as the optimized ARDB [15], customized arsenic resistance database [17] and nitrogen-metabolism genes database extracted from KEGG.

## Materials and Methods

### Datasets used in the tests

The six tested datasets came from three samples, i.e. influent (INF), activated sludge (AS) and anaerobic digestion sludge (ADS) collected from Shatin wastewater treatment plants in Hong Kong (There is no specific permission required for the collection of samples. This sampling site is located at N 22°24′, E 114°12′, Hong Kong, and the field studies did not involve any endangered or protected species). These samples covered a wide range of abundances of the target genes tested in this study. Influent was a composite sample from three influent samples collected in November and December in 2011 and January in 2012. AS was collected from an aeration tank in March 2012 and ADS was collected from an anaerobic digester in March 2012. The details of ADS samples could be found in our previous publication [18]. DNA extraction was performed using FastDNA Spin kit for Soil (MP Biomedicals, CA, USA). High throughput sequencing was performed by the Beijing Genomics Institute (BGI, Shenzhen, China) using illumina Hiseq 2000.

ITags were generated from the paired-end reads using a customized python script. In detail, one of the paired-end reads was converted into its reverse-complement counterpart. If the reverse-complement counterpart and the corresponding paired-end read had an overlap longer than 10 bp, these two reads were merged into a longer itag [18]. For each sample, 10 million reads and 10 million itags were used for the evaluation. The test datasets were deposited in MG-RAST with the accession numbers of 4579259.3 (R_AS_1), 4579258.3 (T_AS), 4579255.3 (R_ADS_1), 4579254.3 (T_ADS), 4579256.3 (R_INF_1) and 4579257.3 (T_INF).

### Tools used in the hybrid annotation pipeline

UBLAST is one of the tools in USEARCH (version 7.0). The free version of 32-bit USEARCH was downloaded from http://www.drive5.com/usearch/. Search acceleration setting “-accel” of UBLAST was tested using the value of 0.5, 0.8 and 1. Default termination options (“-maxaccepts” and “-maxrejects”) were used in all of the tests. Potential matched sequences were extracted from the metagenomic dataset using Perl. Version of BLASTX program is 2.2.28+. Commands used in the tests were listed in Text S1.

## Results and Discussion

### Comparison of RAPSearch2 and UBLAST in ARGs-like sequences annotation

The previously reported hybrid approach uses UBLAST as the first identifier for potential matches in the database [16]. RAPSearch2 was also one of the ultra-fast tools in database search and only have a small portion of missed sequences when compared to direct BLASTX [9], [10]. Therefore, we made a comparison of annotation result from RAPSearch2 and UBLAST to evaluate their speed and annotation accuracy first.

All the tests in the present study were performed using 1 thread on a 16-core workstation (Lenovo ThinkStation-D20: CPU 2.40 GHz×16 threads; Memory 96 GB). Six datasets of different sequence lengths (read or merged itag) were used to evaluate the performance of RAPSearch2 and UBLAST by comparing the time consumed and the annotation results against the optimized ARDB which contained 2998 protein sequences with the size of 1.2 million amino acids (aa) [15]. The six datasets came from three samples, including influent (INF), activated sludge (AS) and anaerobic digestion sludge (ADS) from a wastewater treatment plant. Three of the datasets were illumina reads of 100 bp (R_INF, R_AS and R_ADS) and the other three datasets (T_INF, T_AS and T_ADS) were itags (162∼178 bp) merged from the paired-end illumina reads.

UBLAST was 11∼15 times faster than RAPSearch2 (Table 1) in the search of ARGs-like sequences. It only took about 8 min to search 10 million metagenomic reads against the optimized ARDB while RAPSearch2 took 93 min under the same condition. For itags of longer sequence length, it took 11∼14 min to finish the alignment process of 10 million itags by UBLAST, doubling the time used for reads alignment against the optimized ARDB. Searching itags using RAPSearch2 took 164∼177 min for the same search.

**Table 1:** Comparison of UBLAST and RAPSearch2 in ARGs annotation using the optimized ARDB.

Test datasets^a^	Average sequence length/bp	Time consumed/min^b^		Number of ARGs-like sequences^c^			Number of sequences overlap with BLASTX		Number of annotation accession number overlap with BLASTX^d^	
		UBLAST	RAPSearch2	BLASTX	UBLAST	RAPSearch2	UBLAST	RAPSearch2	UBLAST	RAPSearch2
R_INF	100	8	92	6,646	7,011	6,467	6,566	6,301	2,466	1,973
R_AS	100	6	93	322	341	291	308	277	141	95
R_ADS	100	7	93	361	388	347	351	327	127	99
T_INF	162	14	164	6,972	6,970	6,556	6,856	6,465	2,931	2,436
T_AS	167	11	170	254	248	236	241	228	102	98
T_ADS	178	12	177	336	330	304	326	300	134	120

a : Each dataset contained 10 million sequences. ^b^: Both UBLAST and RAPSearch2 were performed using 1 thread in the search against the optimized ARDB. Parameter of sensitivity was set as –accel 0.5 in UBLAST ^c^: ARGs-like sequences were search with E-value of 1e-5, then further identified with sequence identity ≥90% and hit length ≥25 aa. ^d^: The number of sequences which have the same annotation (accession number) with BLASTX within the overlapped sequences between UBLAST/RAPSearch2 and BLASTX.

ARGs-like sequences were first selected using *E*-value cutoff of 1e-5 in the alignment process and then further identified using the cutoff of sequence identity ≥90% and hit length ≥25 aa. The ARGs-like sequences identified using UBLAST and RAPSearh2 were compared with those identified using direct BLASTX. UBLAST searching shared more ARGs-like sequences with BLASTX while the ARGs-like sequences shared by RAPSearch2 and BLASTX were less (Table 1). For example, out of the 6,646 ARGs-like sequences in the sample R_INF identified using BLASTX, 6,566 of them (98.8%) were shared with the UBLAST results, while only 6,301 (94.8%) were shared by RAPSearch2. Similar results were found for the other 5 datasets, demonstrating that ARGs-like sequences obtained using UBLAST may overlap with most (averagely>97.0%) of those obtained by BLASTX, more than those using RAPSearch2. Nevertheless, the annotation results from UBLAST were different from BLASTX, neither were the results from RAPSearch2. Among the 6,566 shared sequences from UBLAST and BLASTX in R_INF, only 2,466 sequences have the same annotation (the same accession number of ARGs sequences). In the shared sequences from RASearch2 and BLASTX, only 1,973 sequences have the same annotation result.

### Steps and cutoff used in the hybrid annotation pipeline

According to the comparison result of RAPSearch2 and UBLAST, UBLAST was preferred for the primary selection of potential matches. Although ARGs-like sequences obtained using UBLAST may overlap with most of those obtained by BLASTX, the detail annotation of ARGs-like sequences using UBLAST was largely inconsistent with that obtained using BLASTX. Among the sequences shared by UBLAST and BLASTX, only 36.2%∼45.8% of them were annotated to the same reference sequence (with the same accession number) in the optimized ARDB database (Table 1). UBLAST uses an index of seeds in the database for the search [11] while BLASTX parses every reference sequence in the database in the search of target sequences. Therefore, UBLAST can achieve ultra-fast database search but may have annotation results different from those obtained by BLASTX and need to be subjected to BLASTX again for accurate similarity search.

**Figure 1:**
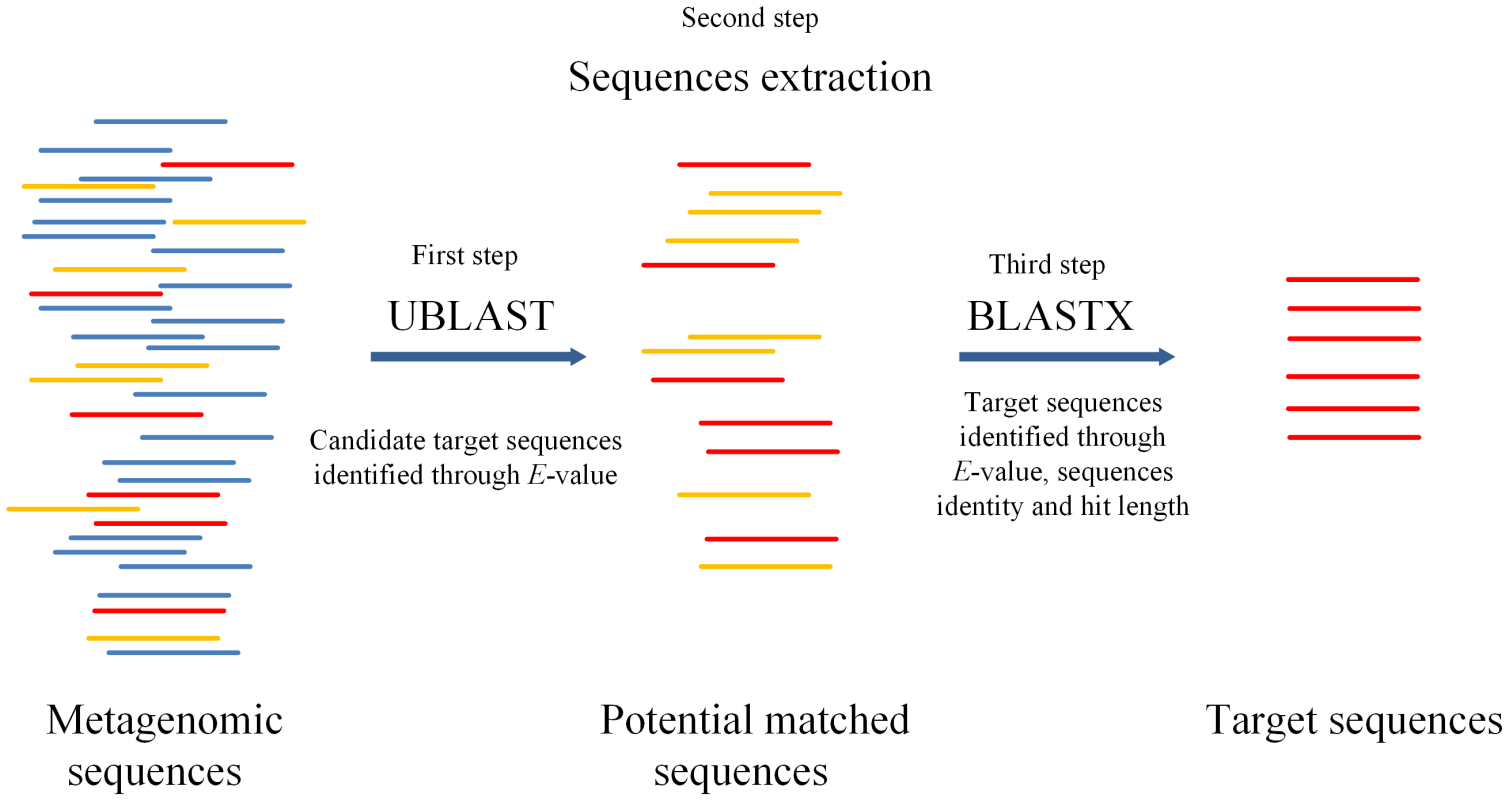
Process of the hybrid annotation pipeline using UBLAST and BLASTX. The potential matched sequences are firstly identified through ultra-fast UBLAST using the cutoff of *E*-value, and then the potential matched sequences are extracted. Further identification and annotation of these potential matched sequences are performed by BLASTX using cutoff of *E*-value, sequence identity and hit length.

The hybrid pipeline included the following steps (Figure 1). The potential matched sequences will be first selected according to the cutoff of *E*-value using UBLAST. *E*-value is the only cutoff used in the selection of potential matched sequences since the application of the other cutoffs, i.e. sequence identity and hit length, would reduce the coverage of target sequences through the selection of UBLAST. These potential matched sequences are then extracted from the metagenomic data and finally subjected to BLASTX using all the cutoffs of *E*-value, sequence identity and hit length for the accurate identification and annotation of the target sequences.

This hybrid annotation pipeline has its unique advantage in the annotation of metagenomic sequences using small specific protein database, such as database of ARGs. The first step of UBLAST can significantly reduce the amount of potential matched sequences which need further BLASTX search for accurate annotation. In the present study, starting with 10 million sequences in each dataset, UBLAST against the optimized ARDB screened out 9,797 to 24,760 potential matched sequences in the datasets of reads and 41,461 to 66,982 potential matched sequences in the datasets of itags, accounting for just 0.1% to 0.7% of the 10 million sequences (Table S1).

The number of potential matched sequences depended on the abundance of the target genes in the sample. For example, ARGs abundance was the highest in influent datasets, having 6,646 ARGs-like sequences using direct BLASTX in R_INF and 6,972 in T_INF. Correspondingly, the number of potential matched ARGs-like sequences obtained using UBLAST were also the highest in influent datasets, i.e. 24,760 potential ARGs-like sequences in R_INF and 66,982 in T_INF. On the contrary, ARGs abundance was the lowest in AS datasets, only 322 and 254 ARGs-like sequences were identified by direct BLASTX in R_AS and T_AS, respectively. Thus the numbers of potential matched ARGs-like sequences in R_AS and T_AS using UBLAST were also the lowest (Table S1).

The comparison of potential matched ARGs-like sequences identified by UBLAST using only the cutoff of *E*-value 1e-5 and ARGs-like sequences using direct BLASTX showed that the potential matched ARGs-like sequences covered over 99.4% of the ARGs-like sequences identified by BLASTX (Table S1), indicating that only a small portion of (<0.6%) target sequences was missed in UBLAST selection, compared to the benchmark using direct BLASTX.

As mentioned, the annotation results using UBLAST and BLASTX were very different, only 36.2%∼45.8% of the target sequences had the same annotation result (the same accession number of the reference sequence in the database) using the optimized ARDB. Therefore, BLASTX was used for the final annotation of selected target sequences in the third step of the hybrid annotation pipeline. After the ultra-fast selection of potential matched sequences by UBLAST, only a small portion of sequences needed to be annotated using BLASTX. The time required for BLASTX was largely reduced and thus the total time required for target functional genes identification and annotation was reduced significantly using the hybrid annotation pipeline.

### Evaluations of time consumed by hybrid annotation pipeline and direct BLASTX

The hybrid annotation pipeline using UBLAST and BLASTX is very efficient in annotating specific functional genes. Taking the annotation of ARGs-like sequences using the optimized ARDB as an example, the hybrid annotation pipeline finished searching of 10 million reads in 8 min and 10 million itags in 14 min, i.e. 90∼177 times faster than direct BLASTX for reads and 44∼67 times for itags (Table 2).

**Table 2:** Time consumed of single BLASTX and the hybrid annotation pipeline.

Data	Database	Single BLASTX*	Hybrid annotation pipeline*			Fold increase in speed
		BLASTX time/min	UBLAST time/min	BLASTX time/min	Total time consumed/min	
R_INF_1	The optimized ARDB (2,998 protein sequences)	1,522	8	9	17	90
R_AS_1		1,597	6	3	9	177
R_ADS_1		1,524	7	3	10	152
T_INF		2,102	14	34	48	44
T_AS		2,276	11	23	34	67
T_ADS		2,320	12	25	37	63
R_INF_1	Arsenic (103,954 protein sequences)	18,969	101	84	185	103
R_AS_1		18,422	82	75	157	117
R_ADS_1		19,086	85	55	140	136
T_INF		28,084	141	349	490	57
T_AS		27,191	133	329	462	59
T_ADS		29,612	147	367	514	58
R_INF_1	Nitrogen_KEGG (63,791 protein sequences)	21,228	89	250	339	63
R_AS_1		22,115	86	244	330	67
R_ADS_1		21,841	85	188	273	80
T_INF		30,528	156	434	590	52
T_AS		30,629	159	437	596	51
T_ADS		33,567	169	445	614	55

*Both BLAST and UBLAST were performed using 1 thread. Sensitivity parameter in UBLAST was set as –accel 0.5.

The comparison of the time required by the hybrid annotation pipeline and BLASTX were conducted using other two much larger functional gene databases, i.e. a database of arsenic resistance genes containing 103,954 protein sequences with total size of 30 million amino acids [17], and a database of genes in nitrogen metabolism containing 63,791 protein sequences with the total sized of 27 million amino acids. The hybrid annotation pipeline was 103∼136 times faster in reads annotation and 44∼67 times faster in itags annotation using the arsenic resistance genes database than direct BLASTX. For the other database of genes in nitrogen metabolism, it was 63∼80 times faster for reads and 51∼55 times for itags.

The time consumed by the hybrid annotation pipeline varied with datasets of different samples, even when the sizes of datasets are the same. It depends on the abundance of target genes in the sample datasets. Between the two tools used, BLASTX is the speed limiting step since it takes much more time than the ultra-fast UBLAST. If there are more potential matched sequences, the time required for BLASTX will increase and consequently the total time required for the hybrid pipeline increases. Among the three kinds of target genes demonstrated in the present study, abundances of ARGs-like sequences had the largest variation, which were 20 times higher in influent (R_INF and T_INF) than in AS and ADS (R_AS, T_AS, R_ADS and T_ADS) (Table S1). Time used by UBLAST for R_INF, R_AS and R_ADS were similar, while time for the following BLASTX for R_INF was about 70% longer than that for R_AS and R_ADS (Table 2).

The time required for direct BLASTX depends on the size of dataset and the size of the reference database. If the sizes of dataset and the database are fixed, the time required for direct BLASTX is fixed, even for different samples. Different from direct BLASTX, the time required for the hybrid annotation pipeline mainly depends on the abundance of target sequences in the original dataset. Thus, this hybrid annotation pipeline will be more favorable for the annotation of specific functional genes, whose abundance is low in the metagenomic dataset.

### Evaluation of annotation accuracy from the hybrid annotation pipeline

The results using the hybrid annotation pipeline were very similar to the results using direct BLASTX, as shown in Table 3. All of the sequences from the hybrid pipeline were included in the results from direct BLASTX in the annotation results using the three different databases. For ARGs annotation, only a small portion of sequences was missed in the hybrid annotation pipeline compared to the results of direct BLASTX. For the dataset R_AS, 320 out of the 322 ARGs-like sequences identified using direct BLASTX could be obtained using the hybrid pipeline, only 2 sequences missed. For the dataset R_ADS, the results using the hybrid pipeline and direct BLASTX were identical. For the dataset R_INF with the highest ARGs abundance, 22 sequences were missed among the 6,646 ARGs-like sequences obtained by direct BLASTX. The missing sequences only accounted for 0.3%. Similar differences were found for itags, only 1 and 2 sequences missed by the hybrid pipeline for the datasets of T_AS and T_ADS, respectively, and 32 (0.5%) ARGs-like sequences missed for the dataset T_INF which had the highest abundance of ARGs. The percentage of the shared ARGs-like sequences from the hybrid pipeline and direct BLASTX were over 99.4% for all tested datasets using the optimized ARDB (Table 3). A dataset of drinking water, which was known to have low microbial diversity [19], was also tested to compare the identification of ARGs using the hybrid approach and the direct BLASTX. The identified target sequences and their annotations are identical for the two different approaches, which proved that the hybrid annotation approach is suitable for identification of the specific functional genes in environmental samples, regardless high or low microbial diversity.

**Table 3:** Difference of annotation results from the hybrid annotation pipeline and direct BLASTX.

Target genes	Dataset	BLASTX^a^	Hybrid annotation pipeline^b^	Number of overlapped sequences	Overlap percentage in direct BLASTX/%	Unique in direct BLASTX	Same annotation	Different annotation
ARGs	R_INF	6,646	6,624	6,624	99.7	22	6,624	0
	R_AS	322	320	320	99.4	2	320	0
	R_ADS	361	361	361	100	0	361	0
	T_INF	6,972	6,940	6,940	99.5	32	6,940	0
	T_AS	254	253	253	99.6	1	253	0
	T_ADS	336	334	334	99.4	2	334	0
Arsenic resistance genes	R_INF	15,240	15,190	15,190	99.7	50	15,189	1
	R_AS	3,132	3,128	3,128	99.9	4	3,128	0
	R_ADS	5,191	5,163	5,163	99.5	28	5,163	0
	T_INF	16,178	16,016	16,016	99.0	162	16,015	1
	T_AS	2,082	2,069	2,069	99.4	13	2,069	0
	T_ADS	5,088	4,998	4,998	98.2	90	4,998	0
Nitrogen metabolism	R_INF	49,408	49,327	49,327	99.8	81	49,327	0
	R_AS	33,674	33,617	33,617	99.8	57	33,617	0
	R_ADS	25,428	25,368	25,368	99.8	60	25,368	0
	T_INF	51,437	51,331	51,331	99.8	106	51,331	0
	T_AS	32,429	32,374	32,374	99.8	55	32,374	0
	T_ADS	23,256	23,135	23,135	99.5	121	23,135	0

a : ARGs-like sequences were search by direct BLASTX with E-value of 1e-5, then further identified with sequence identity ≥90% and hit length ≥25 aa. ^b^: Cutoff of the first step UBLAST was E-value 1e-5. Cutoff of third step BLASTX was E-value of 1e-5, then further identified with sequence identity ≥90% and hit length ≥25 aa.

**Figure 2:**
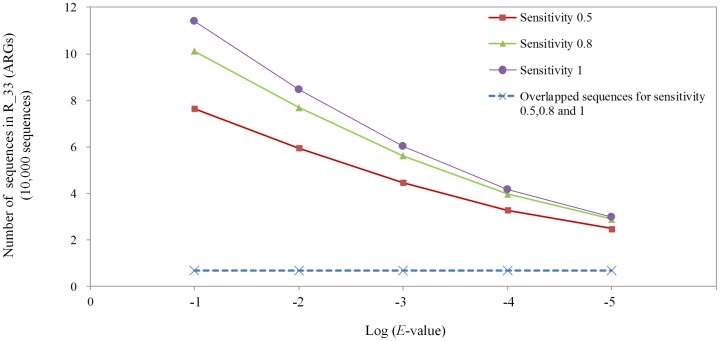
Number of potential ARGs-like sequences and number of overlapped sequences in UBLAST and direct BLASTX. Potential ARGs-like sequences were selected by UBLAST using different E-value cutoff (1e-1, 1e-2, 1e-3, 1e-4 and 1e-5) and different UBLAST sensitivity (-accel 0.5, -accel 0.8 and –accel 1) in dataset R_33.

We tried to increase the percentage of overlapped sequences by loosening the *E*-value cutoff and increasing the search sensitivity in UBLAST. Taking the annotation of ARGs-like sequences as an example, the number of potential matched sequences increases about 3 folds from *E*-value 1e-5 to *E*-value 1e-1 in the dataset R_33, as shown in Figure 2. The increase of UBLAST sensitivity (controlled by –accel parameter in UBLAST) also increased the number of potential matched sequences, but the influence of sensitivity in UBLAST was not as significant as *E*-values. Nevertheless, the percentage of the shared target sequences did not change in ARGs annotation even the highest sensitivity setting in UBLAST and a loose *E*-value cutoff of 1e-1 were used (Table S2) while the analysis time would increase by 4∼6 folds.

The abundances of arsenic resistance gene and genes in nitrogen metabolism were much higher than ARGs in the test datasets. For datasets of reads in the search of arsenic resistance genes, the percentages of the shared annotated sequences between the hybrid pipeline and direct BLASTX were high, ranging from 99.5% to 99.9% in the direct BLASTX. The percentages for shared itags were slightly lower, which were 98.2%∼99.8% (Table 3). For genes in nitrogen metabolism, the percentages of shared annotated sequences were 99.8% for reads and 99.5%∼99.8% for itags (Table 3).

We also tested different *E*-value and search sensitivity in UBLAST for the search of arsenic resistance genes and genes in nitrogen metabolism. Increasing UBLAST sensitivity and *E*-value only added a few new sequences shared by UBLAST and direct BLASTX (Table S2), having little contribution in the total overlapped sequences while time cost was increased by several folds, i.e. 13∼20 folds, and 10∼20 folds for arsenic resistance genes, and genes in nitrogen metabolism, respectively (Table S2).

### Limitation of the hybrid annotation pipeline using UBLAST and BLASTX

This hybrid annotation pipeline is very efficient in the specific functional annotation of large datasets of metagenomic sequences. But it still has a couple of limitations. First, it uses two alignment tools and contains three steps, including the selection of potential matched sequences by UBLAST, extraction of potential matched sequences from the dataset and the final identification and annotation using BLASTX. But this could be easily solved by programming. Second, this hybrid pipeline only has advantage in functional annotation using a specific protein database, and not suitable for the comprehensive functional annotation using large database, such as NCBI-nr. Because the number of potential matched sequences cannot be reduced through the ultra-fast UBLAST when using the comprehensive functional database. Third, this hybrid annotation pipeline would miss some sequences compared with the direct BLASTX, but the portion of these sequences is very low (<1.8%) as demonstrated using three protein databases in the present study.

## Conclusions

The hybrid annotation pipeline was evaluated for specific functional genes annotation in this study. It utilizes the ultra-fast speed of UBLAST to achieve the fast selection of potential matched sequences for subsequent BLASTX, which was proved to be much more efficient compared to direct BLASTX of specific functional genes annotation for metagenomic data. The portion of missed sequences was very small (<1.8%). The application of this hybrid annotation pipeline was demonstrated using six datasets with three protein databases.

## Supporting Information

Table S1 **Comparison of direct BLASTX and UBLAST in ARGs annotation using the optimized ARDB.**
(DOCX)

Table S2 **Tests of different cutoff used in UBLAST on the number of overlapped sequences between UBLAST and direct BLASTX.**
(XLSX)

Text S1 **Command used in the test.**
(DOCX)
