# Supplementary material for: Evaluation of a Hybrid Approach Using UBLAST and BLASTX for Metagenomic Sequences Annotation of Specific Functional Genes
Source: PLoS One. 2014 Oct 27;9(10):e110947. doi: 10.1371/journal.pone.0110947 (PMC4210140; doi:10.1371/journal.pone.0110947)
Supplement: Table S1 — Comparison of direct BLASTX and UBLAST in ARGs annotation using the optimized ARDB. (DOCX) [file pone.0110947.s001.docx]

**Table S1 Comparison of direct BLASTX and UBLAST in ARGs annotation using the optimized ARDB**

| Test datasets | Number of sequences from direct BLASTX^a^ | UBLAST^b^ | | Shared sequences in direct BLASTX and UBLAST | Percentage of shared sequences in BLASTX/% | Annotation accession number overlap with BLASTX in UBLAST | Percentage of overlapped accession number in BLASTX/% |
| --- | --- | --- | --- | --- | --- | --- | --- |
|  |  | Number of sequences | Percentage/%^c^ |  |  |  |  |
| R_INF | 6,646 | 24,760 | 0.3 | 6624 | 99.7 | 2,484 | 37.4 |
| R_AS | 322 | 9,797 | 0.1 | 320 | 99.4 | 143 | 44.4 |
| R_ADS | 361 | 9,904 | 0.1 | 361 | 100 | 131 | 36.3 |
| T_INF | 6,972 | 66,982 | 0.7 | 6,940 | 99.5 | 2,950 | 42.3 |
| T_AS | 254 | 41,461 | 0.4 | 253 | 99.6 | 105 | 41.3 |
| T_ADS | 336 | 46,438 | 0.5 | 334 | 99.4 | 135 | 40.2 |

^a^: Cutoff used in BLASTX were *E*-value 1e-5, 90% sequences identity and 25 aa hit length. ^b^: Cutoff used in UBLAST were *E*-value 1e-5 and detection sensitivity was set as –accel 0.5. ^c^: The percentage of the sequences selected by UBLAST in the 10 million sequences in each tested dataset.
