# Supplementary material for: Evaluation of a Hybrid Approach Using UBLAST and BLASTX for Metagenomic Sequences Annotation of Specific Functional Genes
Source: PLoS One. 2014 Oct 27;9(10):e110947. doi: 10.1371/journal.pone.0110947 (PMC4210140; doi:10.1371/journal.pone.0110947)
Supplement: Text S1 — Command used in the test. (DOCX) [file pone.0110947.s003.docx]

**Text S1 Command used in the test**

BLASTX (version 2.2.28+):

blastx -query Sample.fasta -db database.fasta -evalue 1e-5 -out BLASTX_output_file.txt -outfmt 6 -max_target_seqs 1 -num_threads 1

UBLAST:

./usearch -ublast Sample.fasta -db database.fasta -evalue 1e-5 -accel 0.5 -blast6out UBLAST_output_file.txt -threads 1

Extracting sequences:

1. Extracting sequences ID from the UBLAST result:

awk '{print $1}' UBLAST_output_file.txt > ID_ UBLAST_output_file.txt

1. Extracting sequences from the metagenomic dataset using perl (Script from Edwards Lab https://edwards.sdsu.edu/wordpress/):

perl -ne 'if(/^>(\S+)/){$c=$i{$1}}$c?print:chomp;$i{$_}=1 if @ARGV' ID_UBLAST_output_file.txt Sample.fasta > Extracted_sequences.fasta
